# Genome Sequence of Tilapia Lake Virus Associated with Syncytial Hepatitis of Tilapia in an Ecuadorian Aquaculture Facility

**DOI:** 10.1128/MRA.00084-19

**Published:** 2019-05-02

**Authors:** Kuttichantran Subramaniam, Hugh W. Ferguson, Richard Kabuusu, Thomas B. Waltzek

**Affiliations:** a Department of Infectious Diseases and Immunology, College of Veterinary Medicine, University of Florida, Gainesville, Florida, USA; b Marine Medicine Programme, School of Veterinary Medicine, St. George’s University, St. George, Grenada; KU Leuven

## Abstract

Here, we report the complete coding sequences of tilapia lake virus (TiLV) associated with syncytial hepatitis of tilapia (SHT). The TiLV strain was sequenced from the liver RNA extract of a moribund Nile tilapia (Oreochromis niloticus) fingerling from an Ecuadorian aquaculture facility in 2012.

## ANNOUNCEMENT

Tilapia is a primary source of protein in many developing countries, with an estimated global production of 4.95 million metric tons (estimated value, $10.3 billion) in 2016 (http://www.fao.org/fishery/statistics/global-aquaculture-production/en). Under intensive farming conditions, tilapia succumb to common fish pathogens, including parasites, oomycetes, bacteria, and viruses (1–7). In recent years, tilapia lake virus (TiLV) has emerged as the most important viral threat to the global tilapia industry (8, 9).

Tilapia lake virus was first recorded from a diagnostic investigation of a mortality event involving intensively reared Nile tilapia (*Oreochromis niloticus*) fingerlings in an Ecuadorian aquaculture facility in 2012 (10). The outbreak resulted in mortality of up to 90% in a farm-bred line of tilapia. Clinical signs of disease were observed after transferring the fish into grow-out ponds and included darkening, lepidorthosis, gill pallor, exophthalmia, and abdominal distension due to ascites. Histopathological lesions included necrotizing hepatitis, hepatocellular syncytial cell formation, and necrosis of gastric glandular epithelium and intestinal enterocytes with protein casts (10). These authors suggested that, based on the ultrastructural observation of virus-like particles within hepatocytes, the etiology was viral. They named the disease syncytial hepatitis of tilapia (SHT) (10). A more complete description of the ultrastructural pathological changes and associated viral pathogen followed (11). Subsequently, the full sequence of TiLV was determined from an isolate derived from moribund tilapia in Israel (12). Bacharach and colleagues (12) also reported that the aforementioned tilapia afflicted with SHT from Ecuador were infected with a strain of TiLV with 97.2 to 99.0% nucleotide identity with the 10 coding sequences of the Israeli TiLV isolate. However, the authors did not deposit the Ecuadorian TiLV strain in a public database.

In this study, we determined the 10 coding sequences of Ecuadorian TiLV strain EC-2012, associated with SHT (10). A cDNA library was generated from RNA extracted from the liver of a moribund tilapia involved in the 2012 Ecuadorian mortality event using a NEBNext Ultra RNA library prep kit and sequenced on an Illumina MiSeq sequencer using a MiSeq reagent kit v3 (600-cycle). *De novo* assembly of the 1,695,950 paired-end reads, with an average read length of 263 bp, was performed in SPAdes v3.10.0 (13). BLASTX searches of the assembled contigs against the National Center for Biotechnology Information (NCBI) nonredundant protein database recovered the complete coding sequences of the 10 TiLV segments. The genome was annotated in CLC Genomics Workbench v12.0 with default parameters, and the quality of the assembly was determined by mapping the reads back to the complete coding sequences using Bowtie 2 (14) with default parameters. The average coverage of the TiLV genome was 288 reads/nucleotide and ranged between 47 and 1,603 reads/nucleotide for each segment. BLASTN analysis of the complete coding sequences revealed the highest nucleotide identities (97.4 to 100%) with TiLV strains (AD-2016 and Til-4-2011) from Israel.

A 2012 report of SHT in Ecuador presents one of the earliest confirmed cases of TiLV (10). Our study provides the first complete coding sequences for a TiLV strain from South America. We expect these data will be important in future phylodynamic studies aimed at understanding the origin and global temporospatial spread of TiLV.

### Data availability.

The genome sequences of TiLV strain EC-2012 and the raw sequence data have been deposited in the NCBI GenBank and Sequence Read Archive databases under the accession no. MK392372 through MK392381 and SRX5513850, respectively.
